# Charcot neuroarthropathy patient education among podiatrists in Scotland: a modified Delphi approach

**DOI:** 10.1186/s13047-018-0296-8

**Published:** 2018-09-24

**Authors:** Benjamin Bullen, Matthew Young, Carla McArdle, Mairghread Ellis

**Affiliations:** 1 NHS Lothian Diabetes Foot Service, New Royal Infirmary of Edinburgh, Edinburgh, UK; 2 Podiatry Department, School of Health Sciences, Queen Margaret University, Edinburgh, UK

**Keywords:** Charcot neuroarthropathy, Consensus, Delphi methodology, Diabetic peripheral neuropathy, Metaphor, Patient education, Remission

## Abstract

**Background:**

This evaluation sought to determine current Charcot neuroarthropathy (CN) diabetes patient education practices among Scottish National Health Service (NHS) and academic podiatrists and evaluate novel visual tools and develop expert consensus for future practice.

**Methods:**

Questionnaires collected mixed qualitative and quantitative responses, analysed concurrently within a convergence coding matrix. Delphi methodology permitted member-checking and agreement of consensus over two rounds.

**Results:**

Fourteen participants (16.28%) completed a Round One questionnaire, leading to the generation of four themes; *Experience*; *Person-Centred Care* and the *Content* and *Context* of CN patient education. Seven consensus statements were subsequently developed and six achieved over 80% agreement among 16 participants (18.60%) with a Round Two questionnaire. Respondents agreed CN patient education should be considered for all ‘At-risk’ individuals with diabetic peripheral neuropathy (DPN). Verbal metaphors, including the ‘rocker-bottom’ foot, soft or brittle bones, collapsing, walking on honeycomb and a shattering lightbulb were frequently employed. Visual tools, including visual metaphors and *The Charcot Foot Thermometer*, were positively evaluated and made available online.

**Conclusions:**

Key findings included respondent’s belief that CN education should be considered for all individuals with DPN and the frequent use of simile, analogy and metaphor in CN education. The concept of ‘remission’ proved controversial due to its potential for misinterpretation.

**Electronic supplementary material:**

The online version of this article (10.1186/s13047-018-0296-8) contains supplementary material, which is available to authorized users.

## Background

Charcot neuroarthropathy (CN) is a neuropathic diabetes complication, defined by, typically painless, fractured and dislocated foot bones [1]. Reported among 0.1–0.4% of the diabetes population [2–5], CN prevalence may increase to 10% among individuals with diabetic peripheral neuropathy (DPN) [6]. A lack of pain and limited public and medical practitioner awareness [7] may lead to delayed diagnosis and ineffective offloading, precipitating classic CN midfoot deformity [8], diabetes foot ulceration [9, 10], infection [11] and limb loss [12].

Podiatrists may enlist metaphors, such as the ‘rocker-bottom’ foot, to improve patient appreciation of CN deformity and novel visual tools have been developed to support preventative education [13]. The authors are unaware of previous research investigating podiatrist’s CN patient education practices or the use of metaphor or visual tools to support education. Given an absence of Level One clinical evidence [14] or guidance concerning ‘At-risk’ education, an evaluation was designed to determine current CN patient education practices among NHS and academic podiatrists in Scotland, evaluate novel visual tools and develop expert consensus to inform future practice.

## Methods

A modified Delphi approach was designed to achieve ‘expert consensus’ with anonymous questionnaires over two rounds. Delphi methodology is popular among health researchers, involving focussed surveys and sharing of collated responses before subsequent re-polling [15, 16]. A five-stage approach was adopted, involving problem definition, giving everyone the problem, response collation, giving everyone the collection and repetition [17]. Following approval from Queen Margaret University’s (QMU) Research Ethics Panel, all podiatrists at a single Scottish NHS Trust (*n* = 79) and Lecturers in Podiatry holding honorary contracts with this organisation (*n* = 7) were invited to participate in this research.

A letter describing Delphi methodology accompanied a 55-item Round One questionnaire (Additional file 1: Appendix S1). Respondents were informed that submission of completed study materials constituted consent for participation in, and publication of, this research in line with NHS Health Research Authority (HRA) guidance [18]. Nine quantitative questions concerned podiatrist’s experience while 41 questions addressed the content and frequency of patient education for individuals with diabetes or DPN and those ‘In Remission’ from, or with active, CN. Quantitative responses were captured with a 5-point Likert scale, ranging from *Never* to *Always*.

Five further open-ended questions allowed in-depth qualitative responses concerning how and to whom patient education is targeted and the use of visual tools and metaphors. Novel visual tools, including visual metaphors demonstrating CN pathology and management, and *The Charcot Foot Thermometer,* were also evaluated. These materials may be viewed online [13]. Qualitative Framework Analysis of free text responses was undertaken in the manner described by Gale and colleagues [19] and facilitated with *NVivo 10* software [20]. Round One responses were anonymised, summarised and shared with all NHS and academic podiatrists. Mixed-methods data analysis informed development of seven Consensus Statements, subsequently reviewed throughout Round Two. Achievement of consensus was defined as greater than 80% agreement with each statement [21].

## Results

**Fig. 1:**
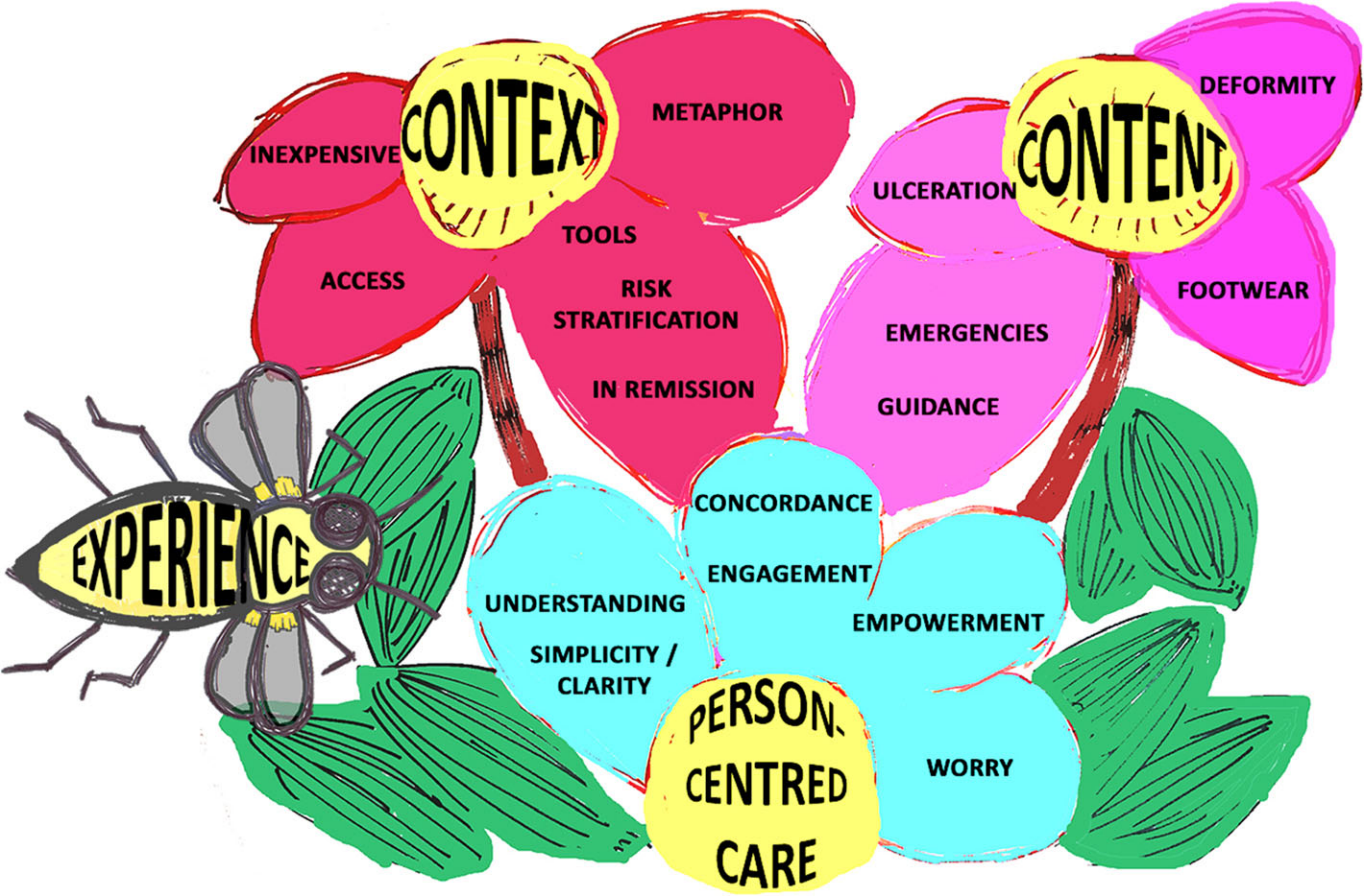
Round One *Flowers and Bee* Analytical Framework, representing *Experience*, *Person-Centred Care* and the *Content* and *Context* of CN patient education

Fourteen respondents (16.28%) completed the Round One questionnaire and anonymised quantitative and qualitative responses were compared within a convergence coding matrix as described by O’Cathain and colleagues [22]. Twenty coding labels emerged and were transformed into a tree diagram [19] (Additional file 1: Appendix 2). Further categorisation lead to the development of a floral arrangement and identification of three themes: *Person-Centred Care* and the *Content* and *Context* of CN patient education (Additional file 1: Appendix 3). These themes, together with *Experience*, were arranged as a *Flower and Bee* visual metaphor, forming the analytical framework (Fig. 1).

All respondents were involved in diabetes foot education and were typically very experienced, practicing for a mean of 15.4 years (range 1–31 years), and predominantly representing Agenda for Change Bands Five and Six (38% Band 5, 54% Band 6 and 8% Band 7). Just over half (54%) of respondents were involved in CN management, with chronic disease seen more commonly. Thirteen respondents were NHS podiatrists and one was a Lecturer in Podiatry. Education appeared to be stratified by risk and metaphors and visual tools, such as leaflets, radiographs and models, frequently employed.

Summarised Round One results were member-checked with all NHS and academic podiatrists (Additional file 1: Appendix 4) and a Round Two questionnaire developed and issued to evaluate seven Consensus Statements informed by Round One data analysis. The Round Two questionnaire included 5-point Likert scale responses, ranging from *Strongly Disagree* to *Strongly Agree*, for each statement with space allowed for qualitative responses (Additional file 1: Appendix 5). Sixteen respondents (18.60%) completed the Round Two questionnaire between January and February 2017, resulting in over 80% agreement for six of seven (85.71%) statements. All 16 respondents replied to five statements, while 15 of 16 (93.75%) respondents replied to statements concerning offloading and *The Charcot Foot Thermometer*. All Consensus Statements, with respective levels of agreement, are included as Table 1.

**Table 1:** Seven consensus statements

	Consensus statement	No. in agreement (%)
1	Charcot foot patient education should be considered for all service users with peripheral neuropathy.	14/16 (87.5%)
2	The term ‘In Remission’ may be applied to those with previous Charcot foot.	10/16 (62.5%)
3	Tools are considered helpful in Charcot foot patient education. Leaflets are issued and x-rays and foot skeleton models are used, when available.	15/16 (93.75%)
4	Metaphors are routinely employed, including the ‘rocker bottom’ foot, soft or brittle bones, ‘collapsing,’ ‘walking on honeycomb’ and ‘like a lightbulb shattering.’	16/16 (100%)
5	Simile, analogy, or metaphor may help when discussing the role of offloading in acute Charcot foot, including prescription glasses as they only work when worn, and a broken leg.	14/15 (93.33%)
6	Visual metaphors show promise in supporting Charcot foot patient education.	13/16 (81.25%)
7	*The Charcot Foot Thermometer* shows promise in demonstrating progress and promoting empowerment and engagement throughout acute Charcot foot management.	13/15 (86.66%)

Fourteen of 16 respondents (87.5%) agreed CN education should be prioritised by risk, including eight (50%) who strongly agreed with the statement*, “Charcot foot patient education should be considered for all service users with peripheral neuropathy.”* Two respondents (12.5%) disagreed. Qualitative responses further suggested many podiatrists favoured delivering CN education to all individuals with DPN, without further risk stratification with one participant stating *“as a result of your initial survey, I am more likely to discuss possible Charcot development with patients developing neuropathy much earlier rather than when it happens now.”* This is consistent with a Round One finding that six of 14 respondents (42.86%) never or rarely discussed CN with individuals with DPN. All 16 respondents agreed metaphors were routinely employed in CN education, including the ‘rocker-bottom’ foot, soft or brittle bones, ‘collapsing,’ ‘walking on honeycomb’ and ‘like a lightbulb shattering.’

Fourteen of 15 respondents (93.33%) agreed or strongly agreed simile, analogy or metaphor may help when discussing the role of offloading in acute CN, including prescription glasses, as they only work when worn, and a broken leg requiring prolonged casting. Thirteen of 16 respondents (81.25%) agreed visual metaphors showed promise in CN patient education while 13 of 15 respondents (86.66%) agreed *The Charcot Foot Thermometer* showed promise in demonstrating progress and promoting empowerment and engagement throughout acute CN management. This visual tool permits patient collection of absolute foot temperatures and discrepancies, routinely assessed at each clinical review.

The only Consensus Statement not meeting the agreement threshold concerned the term ‘In Remission’ for individuals with consolidated CN. While ten (62.5%) respondents agreed or strongly agreed with this terminology, three respondents (18.75%) were unsure and three respondents disagreed or strongly disagreed (18.75%) with its use. One respondent stated *“people (practitioners and service users) may not pay attention to it as it is fine just now,”* while another considered *“In Remission could be interpreted incorrectly by patients and clinicians to mean reduced risk.”*

## Discussion

While response rates were low at 16.28% for Round One and 18.60% for Round Two, they are consistent with a 15.80% response rate reported by Kirkwood et al. [23] in their study of the entire nursing workforce of a single Glasgow-based NHS trust. A key finding from this research was respondent’s belief CN education should be considered for all individuals with DPN. This recommendation is not currently reflected within national guidance. Scottish moderate and high-risk patient information and advice leaflets do not mention CN with relevant information reserved for those with active CN and those ‘In Remission’ [24]. A lack of CN materials for ‘At-risk’ individuals with DPN undermines preventative educational strategies and future research is necessary to determine the health literacy and learning needs of individuals with DPN before designing targeted educational materials.

Simile, analogy and metaphor were frequently employed by respondents when delivering ‘At-risk’ CN education and several examples were shared. Caution is advised to avoid misunderstandings. The concept of remission has proved controversial for this very reason. Since undertaking this research, The Scottish Diabetes Group Foot Action Group’s *Diabetic Foot Risk Stratification and Triage Tool* now includes an ‘In Remission’ category denoting *“previous ulceration, amputation or consolidated Charcot”* [25], p. 185. When applied effectively, a remission analogy invites comparison of care provision and recurrence of diabetes foot disease with cancer treatment [26]. While this analogy may improve patient appreciation of the recurrent nature of diabetes foot disease, including CN, Professor Leonard Levy [27] considered this term overly simplistic, a sentiment reflected in this research.

Finally, respondents considered novel visual metaphors and *The Charcot Foot Thermometer* showed promise in supporting future educational strategies and these are now available online [13]. Visual tools, including visual metaphors, may appeal to individuals with visual learning preferences and those with lower literacy and health literacy levels [28]. The latest assessment of the Scottish population revealed 26.7% occasionally struggled with literacy, while 3.6% were severely challenged [29]. While health literacy rates among the Scottish population are not currently known, 43% of people in England are thought to possess insufficient literacy skills to readily understand health information [30]. Providing verbal and visual information may help support these individuals to gain the knowledge and skills required to identify early signs of CN, present to specialist services and concord with management, ultimately improving clinical outcomes.

## Conclusions

This study has achieved consensus among NHS and academic podiatrists, employed by or holding honorary contracts with a single Scottish health board, respectively. Novel findings included podiatrists’ agreement that CN education should be considered for all ‘At-risk’ individuals with DPN and that simile, analogy and metaphor are routinely employed. Novel visual tools were positively evaluated, however, future research is required to determine if such tools improve CN understanding or concordance with management. To increase the validity of these findings and achieve wider Scottish consensus, this Delphi approach could now be repeated with further Scottish health boards.

## Additional file

Additional file 1: **Appendix S1.** Round One Questionnaire. **Appendix S2:** Initial Tree Diagram [19]. **Appendix S3.** Floral Arrangement. **Appendix S4.** Round One Results. **Appendix S5.** Round Two Questionnaire. (ZIP 4585 kb)

## Abbreviations

CN: Charcot neuroarthropathy
DPN: Diabetic peripheral neuropathy
NHS: National Health Service
QMU: Queen Margaret University
