# Supplementary material for: Charcot neuroarthropathy patient education among podiatrists in Scotland: a modified Delphi approach
Source: J Foot Ankle Res. 2018 Sep 24;11:54. doi: 10.1186/s13047-018-0296-8 (PMC6154915; doi:10.1186/s13047-018-0296-8)
Supplement: Supplementary file 1 — Appendix S1. Round One Questionnaire. Appendix S2: Initial Tree Diagram [19]. Appendix S3. Floral Arrangement. Appendix S4. Round One Results. Appendix S5. Round Two Questionnaire. (ZIP 4585 kb) [file 13047_2018_296_MOESM1_ESM.zip › Additional file 1.docx]

**Appendix S1:** Round One Questionnaire.

**CHARCOT FOOT PATIENT EDUCATION QUESTIONNAIRE:**

This questionnaire addresses Charcot foot patient education and should not require more than 20 minutes to complete. Your participation is entirely voluntary and all submissions will be anonymised. Your responses are of great value as little is currently known as to who receives Charcot foot education and how this is delivered. I am also eager to gather opinions concerning the use of visual tools to enhance patient appreciation of this condition. All anonymised responses will be tallied and themes identified before summarised results are reported back for further consideration. This technique constitutes a modified Delphi approach, summarised separately.

Should you wish to participate, please return your completed questionnaire either by:

email: bbullen@qmu.ac.uk

post: Ben Bullen,

Podiatry Department, Inchkeith House

139 Leith Walk, Edinburgh, EH6 8NP

**PART A: PROFESSIONAL ROLE:**

**YEARS OF PODIATRY EXPERIENCE (IN YEARS):**

**AGENDA FOR CHANGE (AfC) BAND (E.G. 5, 6, 7):**

**PROFESSIONAL ROLE (E.G. PODIATRIST, SPECIALIST PODIATRIST):**

**PLEASE INDICATE YOUR RESPONSE TO EACH QUESTION WITH AN [ X ] IN THE APPROPRIATE BOX. YOU MAY ANSWER AS MANY OR AS FEW QUESTIONS AS YOU PREFER.**

|  | **YES** | **NO** |
| --- | --- | --- |
| **I AM INVOLVED IN DIABETES FOOT SCREENING** | **[ ]** | **[ ]** |
| **I AM INVOLVED IN DIABETES FOOT EDUCATION** | **[ ]** | **[ ]** |
| **I AM INVOLVED IN ‘HIGH RISK’ DIABETES FOOT CARE** | **[ ]** | **[ ]** |
| **I AM INVOLVED IN MANAGING DIABETES FOOT ULCERATION** | **[ ]** | **[ ]** |
| **I AM INVOLVED IN MANAGING CHARCOT FOOT** | **[ ]** | **[ ]** |
| **I AM A MEMBER OF A MULTIDISCIPLINARY DIABETES FOOT TEAM** | **[ ]** | **[ ]** |

**PART B: THE FOLLOWING QUESTIONS RELATE TO THE MANAGEMENT OF ALL SERVICE USERS WITH DIABETES:**

**HOW OFTEN DO YOU DISCUSS THE FOLLOWING WITH ALL SERVICE USERS WITH DIABETES?**

|  | **NEVER** | **RARELY** | **SOMETIMES** | **OFTEN** | **ALWAYS** |
| --- | --- | --- | --- | --- | --- |
| **FOOT ULCER RISK** | **[ ]** | **[ ]** | **[ ]** | **[ ]** | **[ ]** |
| **PREVENTATIVE SELF-CARE** | **[ ]** | **[ ]** | **[ ]** | **[ ]** | **[ ]** |
| **FOOTWEAR AND INSOLES** | **[ ]** | **[ ]** | **[ ]** | **[ ]** | **[ ]** |
| **SIGNS OF INFECTION** | **[ ]** | **[ ]** | **[ ]** | **[ ]** | **[ ]** |
| **SIGNS OF CHARCOT FOOT** | **[ ]** | **[ ]** | **[ ]** | **[ ]** | **[ ]** |
| **AMPUTATION RISK** | **[ ]** | **[ ]** | **[ ]** | **[ ]** | **[ ]** |
| **CARDIOVASCULAR MEDICATIONS** | **[ ]** | **[ ]** | **[ ]** | **[ ]** | **[ ]** |
| **CARDIOVASCULAR MORBIDITY** | **[ ]** | **[ ]** | **[ ]** | **[ ]** | **[ ]** |
| **CARDIOVASCULAR MORTALITY** | **[ ]** | **[ ]** | **[ ]** | **[ ]** | **[ ]** |

**THE FOLLOWING QUESTIONS RELATE TO THE MANAGEMENT OF SERVICE USERS WITH DIABETIC NEUROPATHY:**

**HOW OFTEN DO YOU DISCUSS THE FOLLOWING WITH SERVICE USERS WITH DIABETIC NEUROPATHY?**

|  | **NEVER** | **RARELY** | **SOMETIMES** | **OFTEN** | **ALWAYS** |
| --- | --- | --- | --- | --- | --- |
| **NEUROPATHIC SYMPTOMS** | **[ ]** | **[ ]** | **[ ]** | **[ ]** | **[ ]** |
| **CLINICAL FINDINGS** | **[ ]** | **[ ]** | **[ ]** | **[ ]** | **[ ]** |
| **PREVENTATIVE FOOT CARE** | **[ ]** | **[ ]** | **[ ]** | **[ ]** | **[ ]** |
| **FOOTWEAR AND INSOLES** | **[ ]** | **[ ]** | **[ ]** | **[ ]** | **[ ]** |
| **RISK OF FOOT ULCERATION** | **[ ]** | **[ ]** | **[ ]** | **[ ]** | **[ ]** |
| **SIGNS OF INFECTION** | **[ ]** | **[ ]** | **[ ]** | **[ ]** | **[ ]** |
| **SIGNS OF CHARCOT FOOT** | **[ ]** | **[ ]** | **[ ]** | **[ ]** | **[ ]** |
| **AMPUTATION**  **RISK** | **[ ]** | **[ ]** | **[ ]** | **[ ]** | **[ ]** |

**THE FOLLOWING QUESTIONS RELATE TO THE MANAGEMENT OF PEOPLE WITH NEUROPATHIC DIABETES FOOT ULCERATION:**

**HOW OFTEN DO YOU DISCUSS THE FOLLOWING WITH SERVICE USERS WITH NEUROPATHIC DIABETES FOOT ULCERATION?**

|  | **NEVER** | **RARELY** | **SOMETIMES** | **OFTEN** | **ALWAYS** |
| --- | --- | --- | --- | --- | --- |
| **FOOTWEAR AND INSOLES** | **[ ]** | **[ ]** | **[ ]** | **[ ]** | **[ ]** |
| **TEMPORARY FOOTWEAR** | **[ ]** | **[ ]** | **[ ]** | **[ ]** | **[ ]** |
| **BELOW-KNEE OFFLOADING** | **[ ]** | **[ ]** | **[ ]** | **[ ]** | **[ ]** |
| **SIGNS OF INFECTION** | **[ ]** | **[ ]** | **[ ]** | **[ ]** | **[ ]** |
| **SIGNS OF CHARCOT FOOT** | **[ ]** | **[ ]** | **[ ]** | **[ ]** | **[ ]** |
| **AMPUTATION**  **RISK** | **[ ]** | **[ ]** | **[ ]** | **[ ]** | **[ ]** |

**THE FOLLOWING QUESTIONS RELATE TO THE MANAGEMENT OF ALL SERVICE USERS WITH DIABETIC CHARCOT FOOT:**

**HOW OFTEN DO YOU DISCUSS THE FOLLOWING WITH SERVICE USERS WITH ACUTE DIABETIC CHARCOT FOOT? PLEASE DISREGARD THIS SECTION IF YOU DO NOT MANAGE THESE PATIENTS.**

|  | **NEVER** | **RARELY** | **SOMETIMES** | **OFTEN** | **ALWAYS** |
| --- | --- | --- | --- | --- | --- |
| **FOOT ULCER RISK** | **[ ]** | **[ ]** | **[ ]** | **[ ]** | **[ ]** |
| **SIGNS OF CHARCOT FOOT** | **[ ]** | **[ ]** | **[ ]** | **[ ]** | **[ ]** |
| **TEMPERATURE DISCREPANCY** | **[ ]** | **[ ]** | **[ ]** | **[ ]** | **[ ]** |
| **RADIOGRAPHIC CHANGES** | **[ ]** | **[ ]** | **[ ]** | **[ ]** | **[ ]** |
| **FOOTWEAR AND INSOLES** | **[ ]** | **[ ]** | **[ ]** | **[ ]** | **[ ]** |
| **BELOW-KNEE OFFLOADING** | **[ ]** | **[ ]** | **[ ]** | **[ ]** | **[ ]** |
| **SIGNS OF INFECTION** | **[ ]** | **[ ]** | **[ ]** | **[ ]** | **[ ]** |
| **AMPUTATION**  **RISK** | **[ ]** | **[ ]** | **[ ]** | **[ ]** | **[ ]** |
| **RECURRENCE OF CHARCOT FOOT** | **[ ]** | **[ ]** | **[ ]** | **[ ]** | **[ ]** |

**HOW OFTEN DO YOU DISCUSS THE FOLLOWING WITH SERVICE USERS WITH CHRONIC DIABETIC CHARCOT FOOT? PLEASE DISREGARD THIS SECTION IF YOU DO NOT MANAGE THESE PATIENTS.**

|  | **NEVER** | **RARELY** | **SOMETIMES** | **OFTEN** | **ALWAYS** |
| --- | --- | --- | --- | --- | --- |
| **FOOT ULCER RISK** | **[ ]** | **[ ]** | **[ ]** | **[ ]** | **[ ]** |
| **SIGNS OF CHARCOT FOOT** | **[ ]** | **[ ]** | **[ ]** | **[ ]** | **[ ]** |
| **TEMPERATURE DISCREPANCY** | **[ ]** | **[ ]** | **[ ]** | **[ ]** | **[ ]** |
| **RADIOGRAPHIC CHANGES** | **[ ]** | **[ ]** | **[ ]** | **[ ]** | **[ ]** |
| **FOOTWEAR AND INSOLES** | **[ ]** | **[ ]** | **[ ]** | **[ ]** | **[ ]** |
| **BELOW-KNEE OFFLOADING** | **[ ]** | **[ ]** | **[ ]** | **[ ]** | **[ ]** |
| **SIGNS OF INFECTION** | **[ ]** | **[ ]** | **[ ]** | **[ ]** | **[ ]** |
| **AMPUTATION**  **RISK** | **[ ]** | **[ ]** | **[ ]** | **[ ]** | **[ ]** |
| **RECURRENCE OF CHARCOT FOOT** | **[ ]** | **[ ]** | **[ ]** | **[ ]** | **[ ]** |

**PART C: PATIENT EDUCATION FOR ‘AT RISK’ PATIENTS:**

- DO YOU CURRENTLY DISCUSS CHARCOT FOOT WITH DIABETIC SERVICE USERS AT RISK OF DEVELOPING THIS CONDITION, INCLUDING THOSE A WITH HISTORY OF CHARCOT FOOT, NEUROPATHIC FOOT ULCER OR FOOT SURGERY?

| **PLEASE COMMENT HERE:** |
| --- |

**ON SUSPICION OF CHARCOT FOOT:**

- HOW IS A POSSIBLE DIAGNOSIS OF CHARCOT FOOT DISCUSSED WITH PATIENTS?
- ARE ANY TOOLS EMPLOYED TO IMPROVE PATIENT APPRECIATION?
- VISUAL TOOLS MAY INCLUDE SHOWING THE PATIENT AN X-RAY, FOOT SKELETON OR MODEL. DO YOU SHOW THE PATIENT PHOTOGRAPHS OR DRAW DIAGRAMS?
- DO YOU CURRENTLY USE METAPHOR TO IMPROVE PATIENT APPRECIATION?
- METAPHORS MAY INCLUDE A *“ROCKER BOTTOM”* OR *“BOAT SHAPED”* FOOT TO DESCRIBE DEFORMITY OR *“BURN OUT”* FOR RESOLUTION. DO YOU DESCRIBE THE BONES AS *“SOFT?”*

| **PLEASE COMMENT HERE:** |
| --- |

**OFFLOADING:**

- HOW IS OFFLOADING OF ACUTE CHARCOT FOOT DISCUSSED? DO YOU EMPLOY SIMILE, ANALOGY OR METAPHOR TO IMPROVE PATIENT APPRECIATION OF THE ROLE OF BELOW-KNEE OFFLOADING?
- EXAMPLES MAY INCLUDE LIKENING A BELOW-KNEE DEVICE TO PRESCRIPTION GLASSES,

I.E. THEY ONLY WORK WHEN WORN.

| **PLEASE COMMENT HERE:** |
| --- |

**PART D: VISUAL TOOLS:**

- **PLEASE TAKE A MOMENT TO CONSIDER THE WEBPAGE:** [**http://redhotfoot.webs.com/charcot-foot**](http://redhotfoot.webs.com/charcot-foot)
- THIS WEBPAGE PRESENTS A SERIES OF VISUAL METAPHORS AIMED AT IMPROVING PATIENT APPRECIATION OF CHARCOT FOOT. I WOULD BE VERY GRATEFUL FOR YOUR OPINION REGARDING THIS PRESENTATION AND THE VISUAL REPRESENTATIONS USED.

| **PLEASE COMMENT HERE:** |
| --- |

**THE CHARCOT FOOT THERMOMETER:**

- **PLEASE TAKE A MOMENT TO CONSIDER THE WEBPAGE:** [**http://redhotfoot.webs.com/charcot-foot-thermometer**](http://redhotfoot.webs.com/charcot-foot-thermometer)
- THE CHARCOT FOOT THERMOMETER IS A TOOL FOR PATIENTS TO RECORD TEMPERATURE DISCREPANCIES BETWEEN THEIR FEET OVER TIME. I WOULD AGAIN BE VERY GRATEFUL FOR YOUR OPINION CONCERNING THE APPLICABILITY OF SUCH A TOOL TO IMPROVE PATIENTS ENGAGEMENT AND CONCORDANCE WITH ACUTE CHARCOT FOOT MANAGEMENT.

| **PLEASE COMMENT HERE:** |
| --- |

**Appendix S2:** Initial Tree Diagram [19].

**Appendix S3:** Floral Arrangement.

**Appendix S4:** Charcot Foot Patient Education Questionnaire Round One Results.

**CHARCOT FOOT PATIENT EDUCATION QUESTIONNAIRE:**

**ROUND ONE RESULTS**

**Experience:**

All respondents were involved in diabetes foot education and were typically very experienced, practicing for a mean of 15.4 years (range 1 – 31 years), and predominantly representing AfC bands five and six (38% band 5, 54% band 6, and 8% band 7). 54% of respondents were involved in Charcot foot management, with chronic disease seen more commonly. 13 respondents were NHS XXXX podiatrists and one was an XXXX Lecturer in Podiatry.

**Person-centred care:**

Several respondents expressed concerns about causing service users to worry. This was supported by quantitative data with amputation risk only discussed often (7%) or always (14%) among all service users, increasing to 21% for both categories when neuropathy was also present. Cardiovascular morbidity and mortality were also infrequently discussed, with 21% never discussing this among service users with diabetes. Issues of engagement and empowerment were frequently raised and preventative self-care was always discussed with 86% of all service users with diabetes. Several respondents felt the Charcot Foot Thermometer would promote empowerment and engagement with treatment by demonstrating progress and potentially enhancing concordance with offloading. The importance of concordance with offloading was further demonstrated by discussion of footwear and insoles either often or always among almost all service user groups. Most respondents found the visual metaphors presented to be simple, clear and readily understandable. Not all respondents found this tool to be simple, however, with one person considering these images ‘messy’, ‘a bit childish’, and not following a journey.

**Content:**

Charcot foot was most commonly discussed in the presence of acute or chronic disease and several respondents requested further guidance as to the content to be addressed. Deformity was discussed within the context of offloading and a ‘rocker bottom’ foot was frequently described. Several respondents reported a ‘focus on footwear,’ however, quantitative results suggest this is more prominent among those with neuropathic foot ulceration. 29% of respondents always discussed footwear and insoles among all service users, increasing to 43% in the presence of neuropathy and 71% with neuropathic foot ulceration.

Several respondents mentioned the importance of emergency care and the provision of appropriate contact details. Ulceration risk was discussed often (64%) or always (29%) among all service users with diabetes, increasing to 36% and 64% respectively when neuropathy was present. Signs of infection were discussed often or always with service users with diabetes among 93% of respondents, independent of whether neuropathy was also present. This contrasts with signs of Charcot foot, which were only discussed often for both groups among 36% of respondents and was rarely if ever always discussed. Participants stated “I have historically always focused on ulcer prevention” and “I usually speak about risk of ulceration, which is the stage on the road to amputation rather than scaring them.”

Signs of Charcot foot and temperature discrepancy were the most commonly discussed topics among service users with acute Charcot foot, being discussed sometimes among 20% and always among 80% of NHS XXXX podiatrists. Signs of Charcot foot and foot ulcer risk were the most commonly discussed topics among service users with chronic disease, being discussed often among 17% and always among 83% of NHS XXXX podiatrists. Temperature discrepancy, footwear, and insoles are also always discussed among 67% of service users with chronic Charcot foot.

**Context:**

Quantitative and qualitative responses suggest signs of Charcot foot are routinely discussed when there is a history of this condition, less frequently among those with neuropathic foot ulceration, and variably with neuropathy, history of foot surgery, or diabetes alone. These findings suggest a level of risk stratification. Patient education leaflets are issued, x-rays and foot skeleton models are used when available, and pictures are used and may be sourced online, however, time limitations may be a barrier. Several respondents were unable to access online visual tools on the <http://redhotfoot.webs.com> website.

Metaphor was frequently employed, most commonly the rocker bottom foot and soft or brittle bones. While most participants used the term ‘soft,’ ‘broken,’ ‘walking on honeycomb,’ ‘collapsing’ and ‘like a lightbulb shattering’ are also used in practice. ‘Burn out’ was employed for resolution and a ‘bag of bones’ also used. There was some disagreement as to whether simile, analogy or metaphor are helpful in discussing the role of offloading in acute Charcot foot. Examples used included an ice-cream cone metaphor to describe pressure redistribution over a larger surface area in a cast, prescription glasses as they only work when worn, and a broken leg.

Responses to the visual metaphors presented were largely positive with participants stating they liked and would use ‘rocker bottom,’ ‘honeycomb,’ and ‘casting and external support,’ visual metaphors in practice. Constructive feedback specifically addressed formatting and review of the boat-shaped foot and computer mouse images. One respondent felt the mouse metaphor did not fit and another considered a lateral view of an acute Charcot foot should also be included. Suggested changes included enlarging text and changing from a column format. One participant considered the presentation “lacked visual connection between the basic diabetic foot issues (neuropathy) and the risk of developing a Charcot foot.”

**Appendix S5:** Round Two Questionnaire.

**CHARCOT FOOT PATIENT EDUCATION QUESTIONNAIRE:**

**ROUND TWO**

My sincere thanks go to those who participated in round one of this modified Delphi approach. Participation in this follow-up questionnaire is very much appreciated and entirely voluntary. All questionnaires will again be anonymised and should not require more than 10 minutes to complete.

Should you wish to participate, please return your completed questionnaire by 16^th^ February 2017 either by:

email: bbullen@qmu.ac.uk

post: Ben Bullen,

Podiatry Department, Inchkeith House

139 Leith Walk, Edinburgh, EH6 8NP

**HOW MUCH DO YOU AGREE WITH THE FOLLOWING STATEMENTS?**

CHARCOT FOOT PATIENT EDUCATION IS PRIORITISED BY RISK:

1. ACTIVE OR PREVIOUS CHARCOT FOOT
2. HIGH: HISTORY OF NEUROPATHIC FOOT ULCER
3. MODERATE: PERIPHERAL NEUROPATHY

| **STRONGLY DISAGREE** | **DISAGREE** | **DON’T KNOW** | **AGREE** | **STRONGLY AGREE** |
| --- | --- | --- | --- | --- |
| **[ ]** | **[ ]** | **[ ]** | **[ ]** | **[ ]** |
| **PLEASE COMMENT HERE:** | | | | |

THE TERM ‘IN REMISSION’ SHOULD BE APPLIED TO THOSE WITH PREVIOUS CHARCOT FOOT.

| **STRONGLY DISAGREE** | **DISAGREE** | **DON’T KNOW** | **AGREE** | **STRONGLY AGREE** |
| --- | --- | --- | --- | --- |
| **[ ]** | **[ ]** | **[ ]** | **[ ]** | **[ ]** |
| **PLEASE COMMENT HERE:** | | | | |

TOOLS ARE CONSIDERED HELPFUL IN CHARCOT FOOT PATIENT EDUCATION. LEAFLETS ARE ISSUED AND X-RAYS AND FOOT SKELETON MODELS ARE USED, WHEN AVAILABLE. PICTURES ARE DRAWN OR SOURCED ONLINE, HOWEVER, TIME LIMITATIONS MAY BE A BARRIER.

| **STRONGLY DISAGREE** | **DISAGREE** | **DON’T KNOW** | **AGREE** | **STRONGLY AGREE** |
| --- | --- | --- | --- | --- |
| **[ ]** | **[ ]** | **[ ]** | **[ ]** | **[ ]** |
| **PLEASE COMMENT HERE:** | | | | |

METAPHORS ARE ROUTINELY EMPLOYED, INCLUDING THE ‘ROCKER BOTTOM’ FOOT AND SOFT OR BRITTLE BONES. TERMS SUCH AS ‘COLLAPSING,’ ‘WALKING ON HONEYCOMB’, AND ‘LIKE A LIGHTBULB SHATTERING’ MAY ALSO BE HELPFUL. RESOLUTION MAY BE DESCRIBED AS ‘BURN OUT.’

| **STRONGLY DISAGREE** | **DISAGREE** | **DON’T KNOW** | **AGREE** | **STRONGLY AGREE** |
| --- | --- | --- | --- | --- |
| **[ ]** | **[ ]** | **[ ]** | **[ ]** | **[ ]** |
| **PLEASE COMMENT HERE:** | | | | |

SIMILE, ANALOGY, OR METAPHOR MAY HELP WHEN DISCUSSING THE ROLE OF OFFLOADING IN ACUTE CHARCOT FOOT, INCLUDING AN ICE-CREAM CONE METAPHOR TO DESCRIBE PRESSURE REDISTRIBUTION OVER A LARGER SURFACE AREA IN A CAST, PRESCRIPTION GLASSES AS THEY ONLY WORK WHEN WORN, AND A BROKEN LEG.

| **STRONGLY DISAGREE** | **DISAGREE** | **DON’T KNOW** | **AGREE** | **STRONGLY AGREE** |
| --- | --- | --- | --- | --- |
| **[ ]** | **[ ]** | **[ ]** | **[ ]** | **[ ]** |
| **PLEASE COMMENT HERE:** | | | | |

VISUAL METAPHORS SHOW PROMISE IN SUPPORTING CHARCOT FOOT PATIENT EDUCATION.

| **STRONGLY DISAGREE** | **DISAGREE** | **DON’T KNOW** | **AGREE** | **STRONGLY AGREE** |
| --- | --- | --- | --- | --- |
| **[ ]** | **[ ]** | **[ ]** | **[ ]** | **[ ]** |
| **PLEASE COMMENT HERE:** | | | | |

THE CHARCOT FOOT THERMOMETER SHOWS PROMISE AS A USEFUL, INEXPENSIVE TOOL, DEMONSTRATING PROGRESS AND PROMOTING EMPOWERMENT AND ENGAGEMENT THROUGHOUT ACUTE CHARCOT FOOT MANAGEMENT.

| **STRONGLY DISAGREE** | **DISAGREE** | **DON’T KNOW** | **AGREE** | **STRONGLY AGREE** |
| --- | --- | --- | --- | --- |
| **[ ]** | **[ ]** | **[ ]** | **[ ]** | **[ ]** |
| **PLEASE COMMENT HERE:** | | | | |
