# Supplementary figures and images for: Charcot neuroarthropathy patient education among podiatrists in Scotland: a modified Delphi approach
Source: J Foot Ankle Res. 2018 Sep 24;11:54. doi: 10.1186/s13047-018-0296-8 (PMC6154915; doi:10.1186/s13047-018-0296-8)

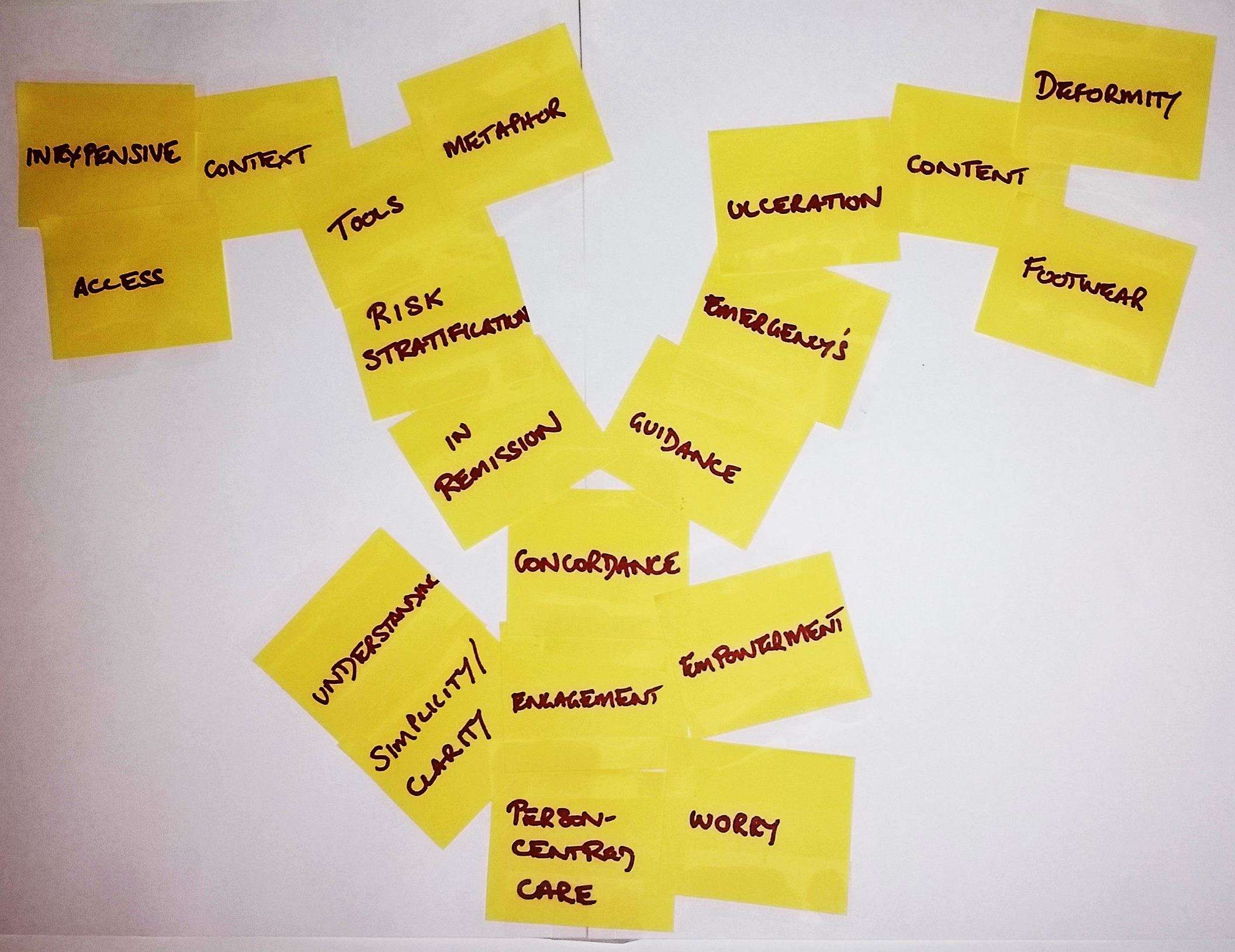

Supplement: Supplementary file 1 — Appendix S1. Round One Questionnaire. Appendix S2: Initial Tree Diagram [19]. Appendix S3. Floral Arrangement. Appendix S4. Round One Results. Appendix S5. Round Two Questionnaire. (ZIP 4585 kb) [file 13047_2018_296_MOESM1_ESM.zip › Appendix 2 Tree DiagramR1.jpg]

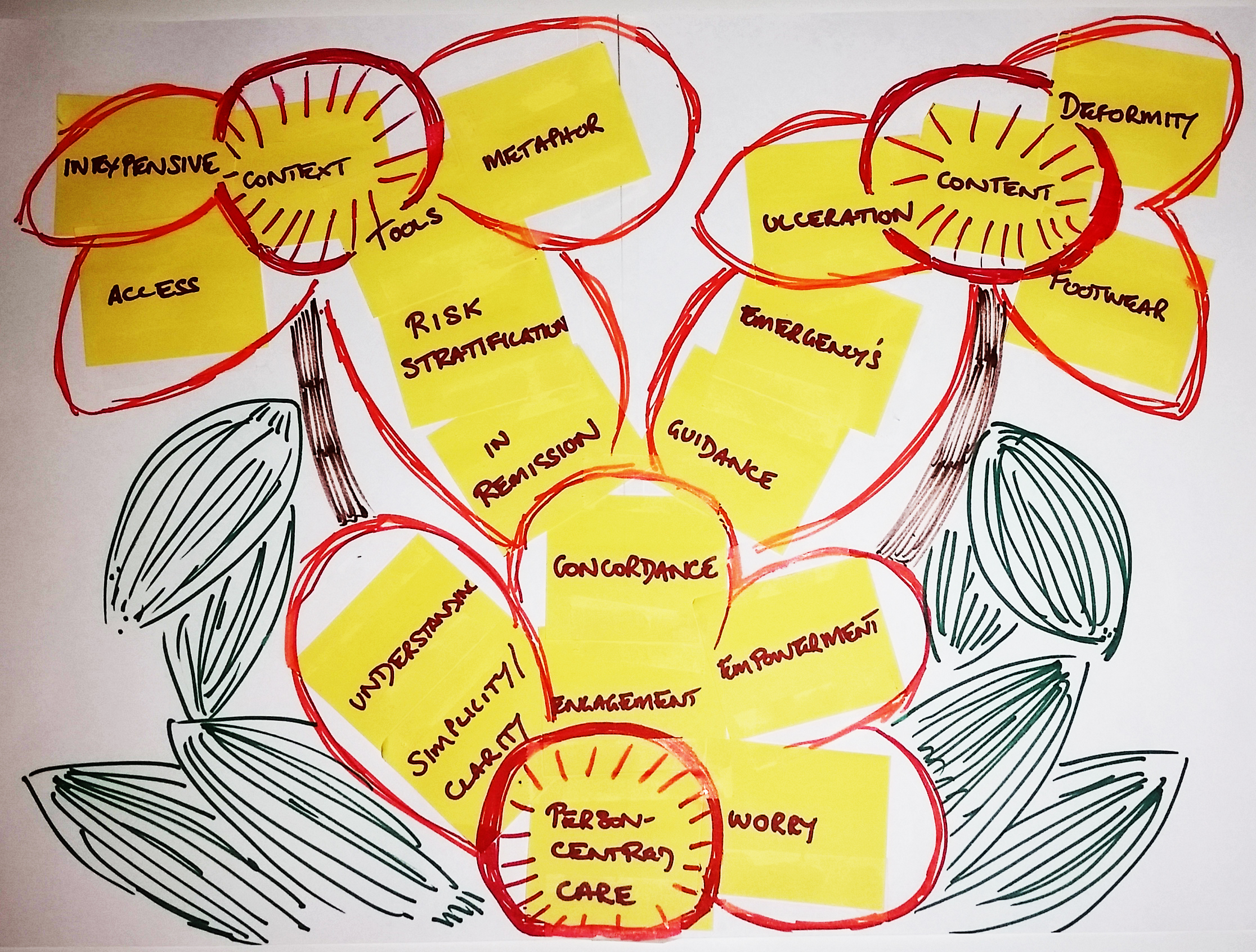

Supplement: Supplementary file 1 — Appendix S1. Round One Questionnaire. Appendix S2: Initial Tree Diagram [19]. Appendix S3. Floral Arrangement. Appendix S4. Round One Results. Appendix S5. Round Two Questionnaire. (ZIP 4585 kb) [file 13047_2018_296_MOESM1_ESM.zip › Appendix 3 Floral ArrangementR1.jpg]
